# A comparative evaluation of the flexural strength and surface hardness of CAD/CAM fabricated and conventional denture bases

**DOI:** 10.1186/s12903-026-07846-1

**Published:** 2026-02-05

**Authors:** Saeed M. AlQahtani

**Affiliations:** Department of Prosthodontics, College of Dentistry, King Khalid University, Abha, 61421 Kingdom of Saudi Arabia

**Keywords:** Flexural properties, Surface hardness, Milled CAD/CAM, Denture base resin

## Abstract

**Objective:**

The objective of this study is to evaluate the flexural strength and surface hardness of denture base resins that have been milled using computer-aided design and computer-aided manufacturing milling (CAD/CAM) and conventional heat-polymerized denture base resins (HP).

**Methods:**

Eighty samples were made using CAD/CAM resin material (Polident CAD/CAM and Interdent CC discs) and conventional heat-polymerised acrylic resin (HP) (IvoBase CAD and PMMA). Based on the denture base material, the samples were categorised into eight groups: each group (*n* = 10), flexural strength (*n* = 40), surface hardness (*n* = 40). Both the surface hardness and flexural strength were evaluated using the Vickers hardness test and the three-point flexure test, respectively. A statistical analysis with a significance level of α = 0.05 was used to analyze the data.

**Results:**

Compared with HP resins, milled CAD/CAM resins demonstrated significantly higher flexural strength and surface hardness (*p* < 0.001). The flexural strength of MHC 72.72 ± 5.95 is significantly lower than that of IHC 78.96 ± 3.36, PCC 108.59 ± 6.51, and VCC 99.88 ± 6.05 MPa. Surface hardness is significantly lower in MHC (18.40 ± 2.28 VHN) than in the other groups: IHC (21.30 ± 2.40 VHN), PCC (31.07 ± 2.07 VHN), and VCC (27.09 ± 1.55 VHN).

**Conclusion:**

Compared with heat-polymerized acrylic resin, milled CAD/CAM resins performed exceptionally well in terms of flexural and surface mechanical properties.

**Supplementary Information:**

The online version contains supplementary material available at 10.1186/s12903-026-07846-1.

## Introduction

Dentures provide a viable treatment alternative for edentulous individuals. Polymethyl methacrylate (PMMA) offers numerous advantages in the fabrication of dentures, including economic viability, favourable physicochemical qualities, and aesthetic appeal [1]. Denture bases are prone to breakage due to extended exposure to physical pressures from chewing. These bases must have adequate resistance and elasticity to endure such stresses without shattering [2]. Flexural strength denotes the maximal twisting force at the fracture threshold, and dentures with elevated flexural resistance provide superior fracture strength. Prolonged flexural stresses from mastication may induce creep, leading to a damping effect and possible deformation [1, 3]. The surface hardness of denture base material is a crucial feature that enhances resistance to surface abrasion, hence minimising microbial colonisation and ensuring colour stability of denture bases. Denture bases must have suitable flexibility and rigidity to avert this issue. Furthermore, acrylic resin’s mechanical properties may be compromised in a humid oral environment due to its rapid moisture absorption [2–4].

Recent advancements in scientific knowledge and technological innovations have introduced digital techniques for denture base fabrication, including CAD/CAM. Digital techniques facilitate the fabrication of a denture base as a single unit and enable the attachment of prefabricated teeth using a suitable glue [5]. Digital technologies enable expedited denture manufacture and streamlined workflows, thereby minimising the likelihood of errors. Digital technology is progressively utilised in dentistry, especially in the milling of dental restorations. CAD/CAM technology facilitates the precise production of dental prostheses, reducing time and patient discomfort relative to conventional procedures; furthermore, it enables the direct replication of an existing denture [4–6]. The milling technique is commonly employed in the fabrication of dentures. The precision of milled dentures depends on the materials used and the milling instruments, specifically the quantity and dimensions of the milling burs. Flexural characteristics are critical factors to consider in denture manufacture, as dentures endure flexural stress during mastication, potentially leading to deformation or fracture over time [5, 7, 8].

In a recent study conducted by Alhotan [9], it was found that the mechanical properties of dental heat-cured acrylic resin were enhanced with silver-doped carbon nanotubes. This could make the denture more resistant to unexpected cracks, scratches, and the invasion of Candida albicans. Abdelraouf [10] found that the flexural strength and water sorption were both enhanced with the addition of 5% weight TiO2 nanoparticles, without compromising the surface micro-hardness or roughness. The results for flexural strength, flexural modulus, impact strength, and surface microhardness were encouraging when self-cured PMMA was reinforced with 0.5% wt silver-doped CNTs, as reported by Hamdy [11]. Zirconia and boron nitride fillers may improve the mechanical properties of self-cured PMMA composites, according to other researchers [11].

Prior research examined the characteristics of HP and milled denture base materials [12]. Nevertheless, research comparing HP and CAD/CAM resins remains scarce. Specific investigations indicated that HP had higher strength than milled samples; however, other studies reported the opposite [13]. A comparable hardness of milled resin to HP was observed, along with an insignificant difference between the two materials [14]. This study assessed the mechanical properties of various denture base materials, including two milling resins and two high-performance resins. This study aimed to use testing to evaluate the flexural strength and surface hardness of several materials for denture base fabrication, emphasizing digital technology (CAD/CAM), and to compare these with heat-polymerized acrylics. The null hypothesis was that there would be no difference in surface hardness and flexural strength between CAD/CAM and HP denture bases.

## Materials and methods

The sample size was determined using a power analysis, with 90% power and a significance level of 0.05, based on other studies [2, 7]. Accordingly, the number of specimens needed was 80 (*n* = 10), as determined by the sample size calculation [2]. Two types of PMMA have been selected for the current study: CAD/CAM and heat-polymerized acrylics for the fabrication of the denture base. Heat-polymerized acrylic resin (HP) (Meliodent and Interacryl) and CAD/CAM materials (Polident and Vipi Block). The samples were categorized into eight groups based on the fabrication of the denture base material (*n* = 10 for each flexural and hardness test, respectively). Table 1 presents a compilation of manufacturers’ materials, types, and processes for denture base construction.

**Table 1:** Type of manufacture and material composition of the denture base resin

S.no.	Material Type	type	Composition	Abbreviation	Manufacturer
1.	Meliodent Heat Cure	Heat-polymerized acrylic resin	Powder: PMMA, benzoyl peroxide Liquid: MMA, ethylene glycol dimethacrylate	MHC	Kulzer GmbH, Hanau, Germany
2.	Interacryl Hot	Conventional; heat-polymerized	PMMA	IHC	Interdent d.o.o.
3.	Polident CAD/CAM disc	disc	PMMA	PCC	Polident d.o.o.
4.	Vipi Block Gum	Discs for milling	Methyl Polymethacrylate, Biocompatible pigments, EDMA, and Fluorescent	VCC	VIPI, São Paulo, Brazil

The surface hardness and flexural strength of eighty samples were measured. The HP discs were fabricated by compression molding. A rectangular wax template was placed in the lower half of the flask, covered with gypsum. After gypsum hardening, a thin layer of separating medium (Cold mould seal Pyrax, Uttarakhand, India) was applied. After applying the gypsum to the upper half of the flask, the flask was sealed with the cover. After the gypsum had set, we removed all of the wax by opening the flask and applying a separating medium to the mould. The packing phase encompassed the positioning and adjustment of mixed HP resin within the mould. The flasks were thereafter positioned in a hydraulic press at 4 bar and transferred to the designated polymerization unit (Dental laboratory polymerizer, REITEL, Feinwerktechnik, Germany) using the flask carrier to maintain pressure. All HPs were made in accordance with the manufacturer’s specifications regarding the polymer-to-monomer ratio and polymerization technique. After polymerization, the flasks were cooled to room temperature. Then, the rectangular acrylic discs were delicately removed from the flask.

CAD/CAM samples were fabricated from the milling blocks using an automated cutting machine (Ivotion Denture System, Ivoclar Vivadent, Liechtenstein) equipped with a diamond blade and polished with 220-grit sandpaper (Brittmoore, Suite 218, Houston, Texas, USA) using a polishing machine (Metaserv 250 grinder-polisher; Buehler GmbH) in wet conditions. The flexural resistance was evaluated using a three-point loading test on a universal testing machine (model EZ 20, Lloyd Instruments Ltd., Fareham, UK), with 10 samples per material tested, measuring 64 × 10 × 3.3 ± 0.2 mm, in accordance with ISO 20795-1:2013. Prior to testing, the samples were submerged in water for 50 ± 10 min at 37 °C [5]. Subsequently, the samples were carefully removed from the water and placed in a parallelogram on the underside of a universal testing machine. The load tension was uniformly increased from 0 at a consistent rate of 5 ± 1 mm/min until the sample fractured. The flexural strength of each sample was quantified using the subsequent formula: FS = 3FL/2bh², where FS denotes flexural strength (MPa), F represents the most significant force exerted on a sample (N), L indicates the distance between the sample supports (mm), b signifies the sample width (mm), and h refers to the sample height (mm). The dimensions of the samples for the hardness test were 15 × 10 × 2.5 ± 0.2 mm. Surface hardness was determined using a hardness tester (QNESS 60 A + EVO, ATM Qness GmbH, Mammelzen, Germany) equipped with a Vickers diamond indenter (25-gf load for 30 s) [5]. Each sample was measured for hardness five times before the average hardness was determined.

### Statistical analysis

All data were entered and analyzed using SPSS version 26. Data normality was assessed using the Shapiro-Wilk test. Because the data were normally distributed, we used analysis of variance (ANOVA) to compare the means of both variables across all groups. We used a multiple-comparison test with the LSD method, as the ANOVA p-value was significant. Data was also presented as error bars with 95% confidence level. P-value ≤ 0.05 was considered significant.

## Results

**Fig. 1:**
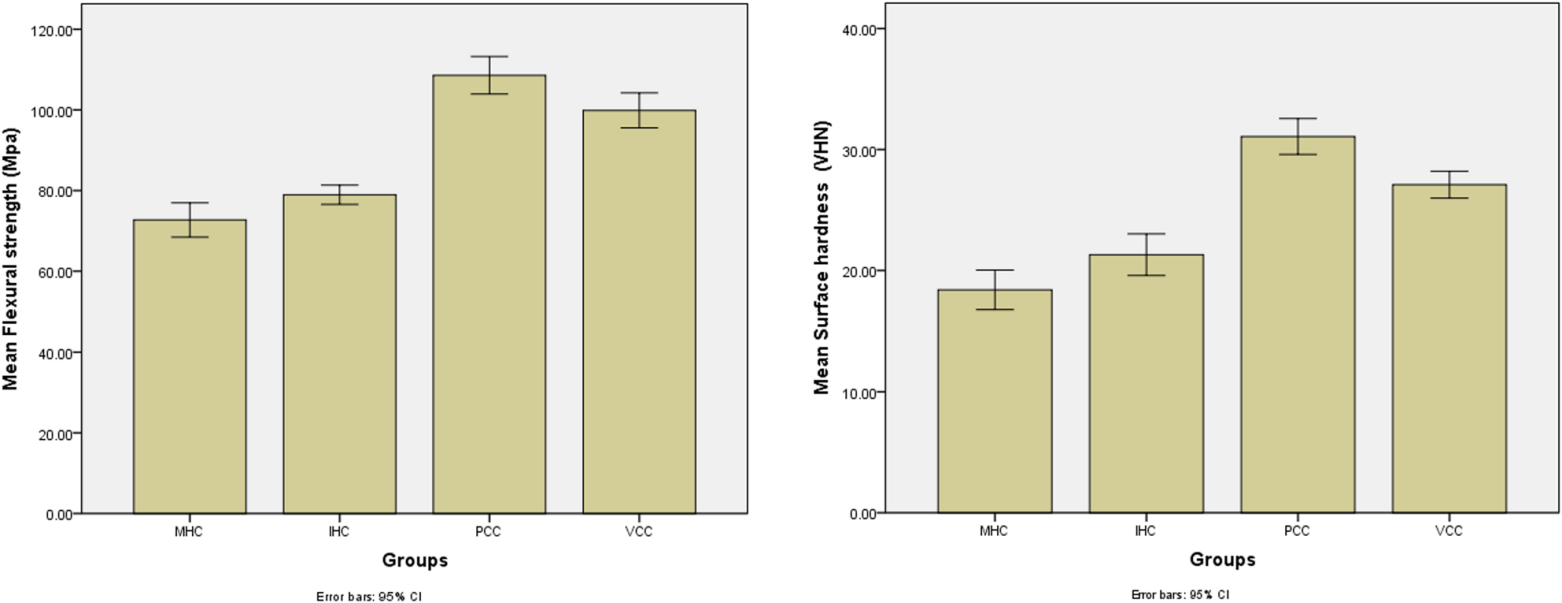
Error bar charts comparing mean flexural strength and surface Hardness of CAD-CAM and conventional denture base materials

The Shapiro-Wilk test for normality shows that the data for both flexural strength and surface hardness in all four groups (MHC, IHC, PCC, VCC) follow a normal distribution, as all p-values are greater than 0.05. This indicates that the assumption of normality holds for these variables. When comparing the mean flexural strength and surface hardness across the groups, significant differences are observed. Table 2 shows that the flexural strength of MHC (Mean = 72.72, SD = 5.95) is significantly lower than that of IHC (Mean = 78.96, SD = 3.36), PCC (Mean = 108.59, SD = 6.51), and VCC (Mean = 99.88, SD = 6.05), with all comparisons showing p-values less than 0.001. This suggests that PCC has the highest flexural strength, followed by VCC, IHC, and MHC. Similarly, surface hardness is significantly lower in MHC (Mean = 18.40, SD = 2.28) compared to the other groups, with PCC (Mean = 31.07, SD = 2.07) and VCC (Mean = 27.09, SD = 1.55) having the highest values. The p-values for all comparisons of surface hardness are also less than 0.001, indicating that the differences are statistically significant. Table 3 shows that multiple-comparison tests further confirm these findings, with all pairwise comparisons revealing significant differences. Specifically, MHC shows significant differences in flexural strength and surface hardness compared with IHC, PCC, and VCC, while PCC consistently shows the highest values for both properties. In conclusion, the results suggest that the type of material (MHC, IHC, PCC, VCC) significantly affects both flexural strength and surface hardness, with PCC superior in both, as shown in Table 4. The error bar charts compare the mean flexural strength and surface hardness of CAD/CAM and conventional denture base materials, with error bars representing ± standard deviation, illustrating differences in mechanical performance and variability between the two groups, as shown in Fig. 1.

**Table 2:** Tests of normality

Groups	Flexural strength (Mpa)			Surface hardness (VHN)		
	Shapiro-Wilk			Shapiro-Wilk		
	Statistic	df	*p*-value	Statistic	df	*p*-value
MHC	0.959	10	0.779	0.951	10	0.679
IHC	0.933	10	0.479	0.932	10	0.466
PCC	0.951	10	0.679	0.983	10	0.979
VCC	0.958	10	0.761	0.949	10	0.655

**Table 3:** Comparison of flexural strength and surface hardness of CAD-CAM and conventional denture base materials

		Mean ± S.D	95% Confidence Interval for Mean		P-value
			Lower Bound	Upper Bound	
Flexural strength (Mpa)	MHC	72.72 ± 5.95	68.46	76.98	< 0.001
	IHC	78.96 ± 3.36	76.56	81.36	
	PCC	108.59 ± 6.51	103.93	113.25	
	VCC	99.88 ± 6.05	95.55	104.21	
	Total	90.04 ± 15.83	84.98	95.10	
Surface hardness (VHN)	MHC	18.40 ± 2.28	16.77	20.03	< 0.001
	IHC	21.30 ± 2.40	19.58	23.02	
	PCC	31.07 ± 2.07	29.59	32.55	
	VCC	27.09 ± 1.55	25.98	28.20	
	Total	24.46 ± 5.39	22.74	26.19	

**Table 4:** Multiple comparison test

	MHC vs.			IHC vs.		PCC vs.
	IHC	PCC	VCC	PCC	VCC	VCC
Flexural strength	0.018	< 0.001	< 0.001	< 0.001	< 0.001	0.001
Surface hardness	0.004	< 0.001	< 0.001	< 0.001	< 0.001	< 0.001

## Discussion

The purpose of this in vitro experiment was to investigate the mechanical properties of denture base materials manufactured using various techniques, with particular emphasis on digital technology (CAD/CAM). Statistical analysis revealed that the study groups varied in flexural strength and surface hardness, therefore rejecting the null hypothesis. The findings of this investigation demonstrated a statistical difference among the various resin categories for all evaluated attributes, leading to the rejection of the study hypothesis.

It is referred to as the material’s flexural strength, modulus of elasticity, bend strength, or transverse strength [15]. Flexure testing is used to determine the stress a material experiences immediately before yielding. Given that a denture base may shatter due to multiple factors, it is essential that its material exhibit strong flexural strength [16, 17]. The findings on the flexural resistance of milled denture bases are inconsistent. A study by Di Fiore [18] yielded conflicting results, indicating that various milled denture materials exhibited flexural strengths comparable to or higher than those of the control heat-polymerized group. Srinivasan [19] and Sahin [20] established that heat-polymerized PMMA has superior flexural strength compared to milled resin materials.

In contrast to the findings of Kamal [21], Chhabra [22], and Selin [23], the results of the current investigation correspond with those of Asli [24], demonstrating that milled materials possess greater flexural resistance than HP denture base resin materials. Considering that milled discs are manufactured at high temperature and pressure using compressed acrylic resin with minimal porosity, shrinkage, or free monomers, the enhanced flexural resistance of milled resins is supported by the current research [25–27]. It is of the utmost importance to acknowledge that variations in the flexural strength values of CAD/CAM and heat-polymerized denture base materials may result from the utilisation of different materials from various manufacturers in the current study.

The study’s results indicate that milled materials exhibit superior flexural properties and surface solidity compared to heat-polymerized resins. Previous research revealed analogous findings regarding flexural characteristics (flexural resistance and modulus) and surface hardness [28]. The elevated flexural characteristics seen in milled samples may be attributed to the distinctive production method of the pre-polymerized discs utilized in denture fabrication. These milled discs are produced under stringent elevated compression and temperature conditions, leading to reduced residual monomer levels and lower plasticizing effects [29, 30]. Contrary to our findings, a specific study reported little change in hardness between milled CAD/CAM and HP [5]. The variance in outcomes in this study may stem from the differing materials examined.

The term “hardness” refers to the capability of a material to withstand localised plastic distortion brought about by mechanical indentation or abrasion [7, 31]. Dentures made from a material with inadequate surface hardness are vulnerable to mechanical brushing, leading to plaque buildup and discolouration, and thereby diminishing their longevity [5, 16]. The current analysis identified two categories of CAD/CAM materials as possessing the maximum surface solidity among the study materials. This observation parallels that of Prpić [5], who documented higher solidity values for CAD/CAM resins than for HP counterparts. Consistent with prior investigations, HP was identified as the resin exhibiting the lowest hardness. Upon relating the flexural resistance and surface solidity of the examined groups, it can be inferred that the mechanical properties of most CAD/CAM materials exceed those of heat-polymerized acrylics [5]. A study by Steinmassl et al. [32] yielded conflicting results, indicating that various CAD/CAM denture base resins exhibited flexural strengths that ranged from comparable to those of the control heat-polymerized group to lower than those of the control heat-polymerized group. Ayman [33] and Pacquet et al. [34]. established that heat-polymerized PMMA exhibits superior flexural strength compared to CAD/CAM denture base material. Unlike the research by Steinmassl et al. [32], Ayman [33], and Pacquet et al. [34], the current study’s findings align with those of Aguirre et al. [17], indicating that CAD/CAM materials exhibit superior flexural strength compared to conventional denture base materials.

The measured surface hardness and flexural resistance data can be elucidated based on the materials’ internal structures [17, 20]. The current study indicates that dentists should acknowledge the availability of various denture base materials with varying mechanical properties, given the advent of digitally generated dentures. The findings of this investigation indicated that the milled resin exhibited enhanced flexural strength, elastic modulus, and surface hardness when juxtaposed with HP, which consistently displayed the least favourable values among all assessed characteristics. Improvements in the chemical composition or manufacturing techniques of CAD/CAM resins are essential to elevate their mechanical properties for clinical applications. This study examined various denture base materials produced through diverse processes, encompassing multiple brands of milled dentures. Subsequent research should examine the characteristics of CAD/CAM resins post-aging to assess their strength in clinical applications. The research exhibited two principal shortcomings. Firstly, oral conditions were absent in the present investigation, and secondly, varied testing environments (dry versus wet) and diverse testing mediums (air or water) were omitted. Both factors may have influenced the outcomes. Future research on new denture base materials should include examinations of flexural modulus, adhesion to synthetic polymer teeth, and residual monomer levels to achieve a more comprehensive understanding. This study examined just specific mechanical qualities. To enhance understanding of the limitations of the tested resins, it is essential to examine factors such as biocompatibility, microbiological adherence, manufacturing accuracy, clinical fit, and color stability. Future research examining the impact of simulated brushing and thermocycling at 10 on various mechanical properties with differing parameters would enhance understanding of the limitations of these materials. Future research should examine the aging characteristics of CAD-CAM denture base materials to assess their durability in clinical applications.

## Conclusions

This in vitro study demonstrates that CAD/CAM denture base materials exhibit enhanced mechanical properties compared with heat-polymerized acrylics. Consequently, both fabrication methods are therapeutically viable, with CAD/CAM materials offering greater durability and long-term performance.

## Supplementary Information

Supplementary Material 1.

## Data Availability

All data generated or analysed during this study are included in this published article.

## Abbreviations

CAD/CAM: computer-aided design and computer-aided manufacturing milling
HP: Conventional heat-polymerized denture base resins
PMMA: Polymethyl Methacrylate
SD: Standard deviation
